# Golgi protein 73 as a biomarker for hepatocellular carcinoma: A diagnostic meta-analysis

**DOI:** 10.3892/etm.2015.2231

**Published:** 2015-01-29

**Authors:** JING YANG, JINGJING LI, WEIQI DAI, FAN WANG, MIAO SHEN, KAN CHEN, PING CHENG, YAN ZHANG, CHENGFEN WANG, RONG ZHU, HUAWEI ZHANG, YUANYUAN ZHENG, JUNSHAN WANG, YUJING XIA, JIE LU, YINGQUN ZHOU, CHUANYONG GUO

**Affiliations:** Department of Gastroenterology, Shanghai Tenth People’s Hospital, Tongji University School of Medicine, Shanghai 200072, P.R. China

**Keywords:** Golgi protein 73, hepatocellular carcinoma, cancer diagnosis, meta-analysis

## Abstract

Hepatocellular carcinoma (HCC) is the most common primary malignancy of the liver and the third leading cause of cancer-related mortality worldwide. Conflicting results have been reported regarding the use of serum Golgi protein 73 (GP73) as a promising serum marker for the diagnosis of HCC; therefore, the aim of the present study was to provide a systematic review of the diagnostic performance of GP73 for HCC. Following a systematic review of the relevant studies, a number of indices associated with the accuracy of the diagnostic performance of GP73, including the sensitivity and specificity, were pooled using Meta Disc 1.4 software. Data were presented as forest plots, and summary receiver operating characteristic (SROC) curve analysis was used to summarize the overall test performance. Eleven studies were included in this meta-analysis. The summary estimates for serum GP73 in diagnosing HCC were as follows: Sensitivity, 77% [95% confidence interval (CI), 75–79%]; specificity, 91% (95% CI, 90–92%); positive likelihood ratio, 4.34 (95% CI, 2.19–8.59); negative likelihood ratio, 0.30 (95% CI, 0.26–0.36) and diagnostic odds ratio, 15.78 (95% CI, 6.95–35.83). The area under the SROC curve was 0.8638, and the Q index was 0.7944. Significant heterogeneity was found. This meta-analysis indicates a moderate diagnostic value of GP73 in HCC; however, further studies with rigorous design, large sample size and multiregional cooperation are required.

## Introduction

Hepatocellular carcinoma (HCC) is one of the most common, aggressive solid malignancies worldwide, accounting for in excess of two-thirds of all primary liver cancer cases (1). Approximately 500,000 new cases of HCC are reported annually, and >75% of these occur in the Asia-Pacific region (2). In the USA, the HCC incidence is increasing at a greater rate than the incidence of any other cancer (3). Furthermore, the five-year survival rate for HCC is <5%, ranking HCC as one of the types of cancer with the worst prognosis (4). As a result, the mechanism underlying the tumorigenesis and the specific measures required for the early diagnosis or effective therapy of HCC are current research focuses (5–8).

HCC commonly arises against a background of chronic liver disease and cirrhosis caused by hepatitis B or C virus (9). In these patients, surveillance strategies for the detection of early HCC are necessary. For >40 years, the most common marker used in clinical practice has been α-fetoprotein (AFP), which is combined with hepatic ultrasonography. AFP is considered to be the gold-standard serum marker for the screening of patients who are at high risk of HCC, as well as for the monitoring of treatment response (10); however, the clinical value of AFP has been questioned due to its low sensitivity and specificity (11). As the overall survival of patients with cirrhosis has improved and the global incidence of HCC has continued to increase, strategies for the early detection of HCC are urgently required (12).

Golgi protein 73 (GP73, otherwise known as Golph2) is a resident Golgi-specific membrane protein that is expressed in the normal liver by biliary epithelial cells. The expression of GP73 undergoes a notable increase in chronic liver diseases, particularly in HCC cells (13). A number of studies have described the use of GP73 as a serum marker for HCC; however, the results have been inconsistent and shown evident heterogeneity (14–16). The aim of the present study, therefore, was to perform a systematic analysis of studies evaluating the diagnostic accuracy of serum GP73 for HCC.

## Materials and methods

### Inclusion and exclusion criteria

Studies were evaluated strictly for their relevance to the selected topic. Eligible studies had to include a representative patient spectrum. The diagnosis of HCC was established by histopathological examination or, if histopathology was not available, by two imaging modalities, such as ultrasound, magnetic resonance imaging or computed tomography, showing a vascular enhancing mass of >2 cm (17). Exclusion criteria comprised studies that evaluated serum GP73 levels by mRNA, DNA or DNA polymorphism analysis and those that did not provide exact values for the sensitivity or specificity of GP73, as well as abstracts, letters, editorials and expert opinions, reviews without original data, case reports and studies lacking control groups.

### Identification of studies

A comprehensive systematic literature review of original investigations into the diagnostic accuracy of GP73 was performed by searching the following electronic databases up to September 2013: PubMed/Medline, Embase, Cochrane Database of Systematic Reviews, Cochrane Central Register of Controlled Trials, Science Citation Index (ISI Web of Science), Chinese Biomedical Literature Database and Chinese National Knowledge Infrastructure (18,18). References from the included studies and any relevant published reports were additionally manually searched. No restrictions were placed on language, study design, year of publication or publisher status. The subject headings and keywords utilized in the search strategy included i) GP73: GP73, Golgi protein 73, Golgi phosphoprotein 2, Golgi membrane protein 1; and ii) HCC: HCC, hepatocellular carcinoma, liver cell carcinoma, hepatic cell carcinoma. No keywords or indexing terms for diagnostic test accuracy were used due to the possibility of relevant studies being missed.

### Study selection

Independent reviews of the studies were performed by two reviewers based on the titles and abstracts, prior to the full texts of any potentially relevant studies being obtained for further assessment. Disagreements between the reviewers were resolved by consensus. If any further study details were required, a request was sent to the authors. When findings from the same patient population were reported by the same author in multiple publications, the most recent or most complete report was identified and used to avoid overlap between cohorts.

### Data extraction

The following data were extracted independently from the included studies by two reviewers: Authors, year of publication, journal, study design, number of patients, type of marker assay, cut-off values and raw data regarding the sensitivity and specificity (number of true-positive, false-negative, true-negative and false-positive results) for comparisons of patients diagnosed with HCC versus controls. Disagreements were resolved through discussion with a third reviewer.

### Assessment of methodological quality

The quality of each study was evaluated according to the Quality Assessment of studies of Diagnostic Accuracy included in Systematic reviews (QUADAS) checklist recommended by the Cochrane Collaboration. Each of the 14 items in the QUADAS checklist was scored as ‘yes’, ‘no’ or ‘unclear’ (20).

### Data analysis

Using Meta Disc software (version 1.4; Clinical Biostatistics Unit, Ramón y Cajal Hospital, Madrid, Spain), the receiver operating characteristic (ROC) plane was drawn and the Spearman correlation coefficient was calculated to estimate if there was a threshold effect. The overall sensitivity, specificity, positive likelihood ratio (PLR), negative likelihood ratio (NLR) and diagnostic odds ratio (DOR) were calculated. Data were presented as forest plots, which showed the results of individual studies with the corresponding 95% confidence intervals (CIs). Summary ROC (SROC) curve analysis was used to summarize the overall test performance. The Midas model for Stata (version 12.0; StataCorp LP, College Station, TX, USA) was used to construct the funnel plots and calculate the P-values. Publication bias existed when P<0.05 was observed. Meta-regression was also performed in an attempt to explain the observed heterogeneity.

## Results

### Study retrieval

**Figure 1:**
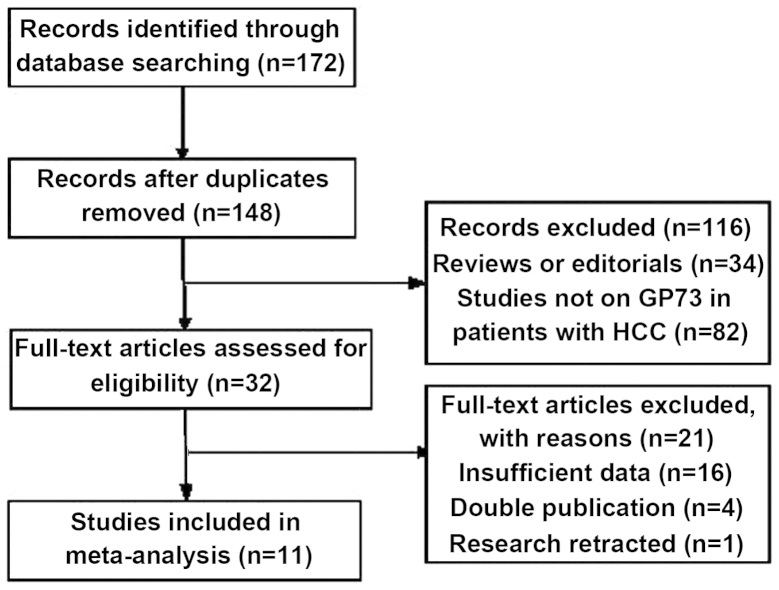
Flow diagram showing the article selection process for the meta-analysis. GP73, Golgi protein 73; HCC, hepatocellular carcinoma.

A total of 172 studies were found and 32 were considered to be eligible for inclusion in the analysis. Following the full-text review, 21 studies were excluded: 16 due to the results not allowing the calculation of sensitivity or specificity; four due to a suspected overlap in the study population or a duplicate publication; and one due to a retraction by the author (21). Finally, 11 studies were available for the meta-analysis. These studies included 6,711 patients who received serum GP73 tests (22–32), 1,887 of whom were diagnosed with HCC by histopathology or two imaging modalities. A flow diagram of the study selection process is shown in Fig. 1.

The characteristics of each study are shown in Table I. The number of patients in each of the 11 studies was >100, with little difference in the characteristics among the studies. The GP73 cutoff values differed substantially, which may have been a source of heterogeneity. The ethnicity in nine studies was Asian.

### Quality of studies

The QUADAS criteria were used to evaluate the quality of the 11 selected studies. As shown in Table II, all the studies fulfilled between seven and 11 of the 14 described criteria. Summary scores were not calculated, as their interpretation can be problematic and potentially misleading (33). All the studies used a retrospective design. In 10 studies, healthy individuals were recruited for the control group; the percentage of HCC diagnoses in these studies ranged between 18.7 and 59.2%. All the studies reported the diagnostic standard of HCC, and four reported the tumor stage of the patients with cancer (23,24,27,28). The serum GP73 levels were interpreted in a blinded manner in only one out of the 11 studies.

### Threshold effect

**Figure 2:**
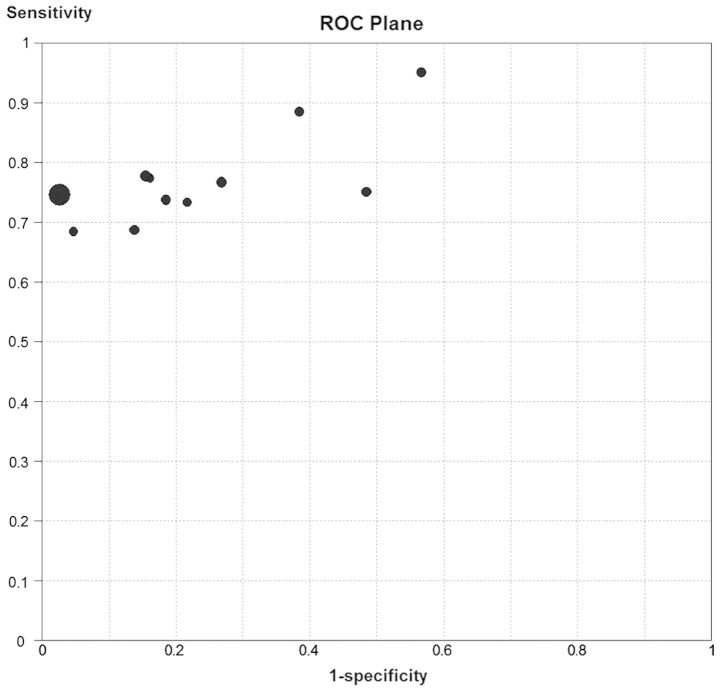
ROC plane output by the Meta Disc 1.4 software. ROC, receiver operating characteristic.

When there is a threshold effect, an inverse correlation is demonstrated between the sensitivity and specificity, leading to a typical ‘shoulder arm’ of the ROC plane distribution. Spearman correlation analysis also suggests a strong positive correlation. In the present study, the ROC plane output by the Meta Disc 1.4 software (Fig. 2) showed a nontypical shoulder arm appearance; the calculated Spearman correlation coefficient value was 0.591 and the P-value was 0.056, suggesting that there was no threshold effect.

### Summary diagnostic accuracy of serum GP73 for HCC

**Figure 3:**
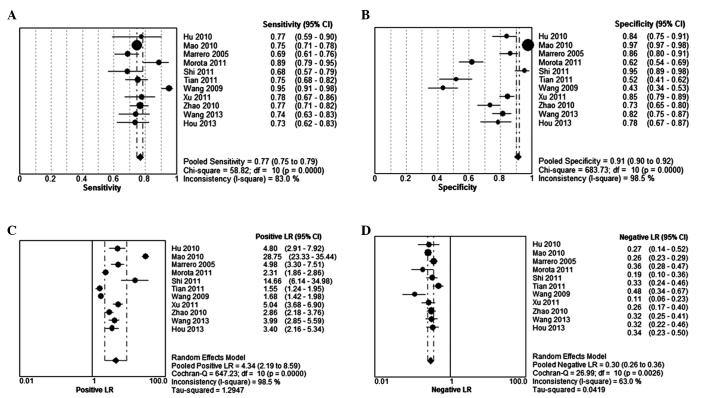
Forest plot of the meta-analysis of each index: (A) Sensitivity; (B) specificity; (C) PLR; (D) NLR. PLR, positive likelihood ratio; NLR, negative likelihood ratio.

**Figure 4:**
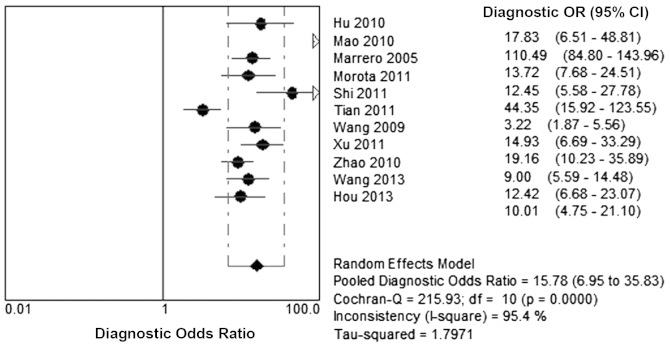
Forest plot of the DOR in each study included. DOR, diagnostic odds ratio.

The DerSimonian-Laird (random effects) model was used to calculate the pooled value. The sensitivity observed ranged between 43 and 88.6% (summary, 77%; 95% CI, 75–79%) (Fig. 3A), while the specificity ranged between 51.8 and 97.4% (summary, 91%; 95% CI, 90–92%) (Fig. 3B); the PLR was 4.34 (95% CI, 2.19–8.59) (Fig. 3C) and the NLR was 0.30 (95% CI, 0.26–0.36) (Fig. 3D). The PLR value indicated that patients with HCC had a 4.3-fold higher chance of a positive GP73 assay compared with patients without HCC. Similarly, the NLR indicated that, if the GP73 assay was negative, the probability of these patients developing HCC was ~30%. Thus, GP73-negative results may not be used to exclude HCC. It was also noted that the summary DOR was 15.78 (95% CI, 6.95–35.83) for GP73 (Fig. 4). The sensitivity, specificity, PLR, NLR and DOR with the 95% CIs for each study were presented in a forest plot, and significant heterogeneity was observed.

**Figure 5:**
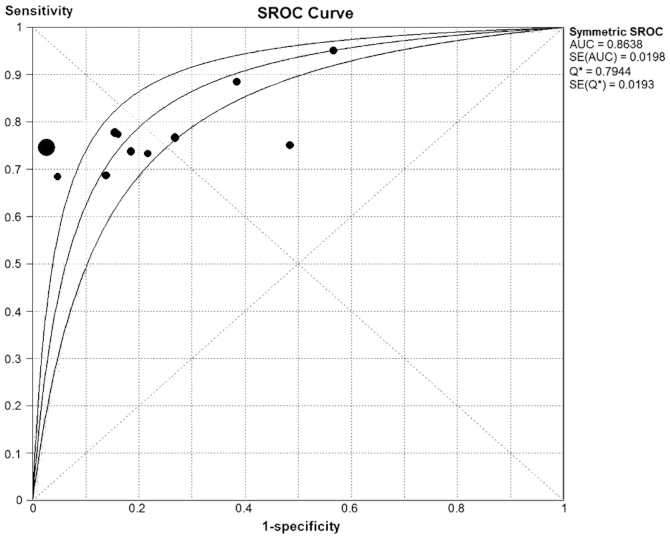
SROC curves for Golgi protein 73. SROC, summary receiver operating characteristic; AUC, area under the curve; SE, standard error.

The SROC approach is the standard strategy for the meta-analysis of the diagnostic reporting pairs of sensitivity and specificity (34). This approach uses DOR as the primary outcome measure, which eliminates the effect of a possible threshold (35). As shown in Fig. 5, the area under the SROC curve was 0.8638, with a standard error of 0.0198 and Q^*^ of 0.7944, suggesting a comparable diagnostic value of GP73 for HCC.

### Meta-regression for heterogeneity

To investigate heterogeneity, attempts were made to explore the following study characteristics using meta-regression: Population characteristics (gender, ethnicity, age, disease type and stage distribution), study design (prospective or retrospective and year of publication) and test characteristics (cutoff value, test type and number of tests per screening round); however, due to the unsatisfactory methodological quality of the studies or incomplete data, year, assay type and country were the only three features examined. The accuracy measure used was DOR, since it was a unitary measure of diagnostic performance that encompassed sensitivity and specificity or PLR and NLR. It was found that the differences among the ethnicities had a significant effect on the DOR (Table III). This may have been due to the fact that the sample size of Western patients was small compared with the number of Asian patients.

### Publication bias

**Figure 6:**
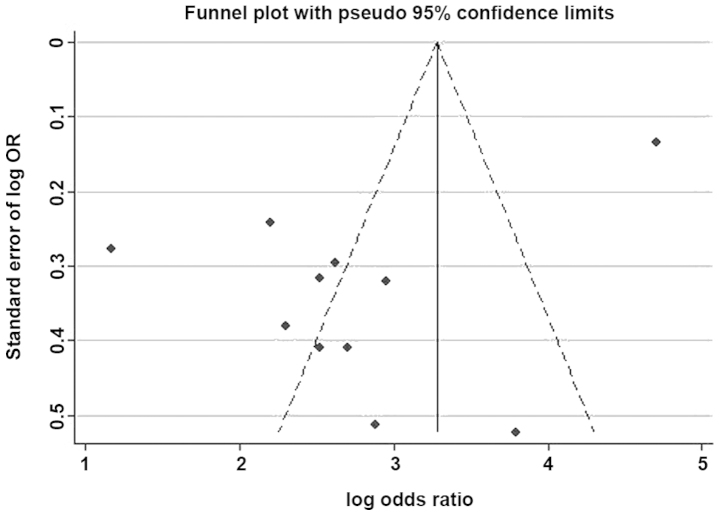
Deeks’ funnel plot created using the ‘metafunnel’ command of Stata (version 12.0).

Deeks’ funnel plot was created using the ‘metafunnel’ command of Stata version 12.0. As shown in Fig. 6, the funnel plot was asymmetrical, which meant a publication bias in our study; however, the most recent studies have tended not to assess publication bias due to the fact that the investigation of reporting and publication bias in diagnostic accuracy studies has been shown to be problematic (36,37). A possible reason is that numerous studies are performed without study registration (36–38), making it impossible for an exact assessment of publication and reporting bias to be performed from registration.

### Sensitivity analysis

In order to investigate the stability of the meta-analysis, sensitivity analysis was performed from three aspects. Firstly, one study at a time was excluded to assess the effect of a single study on the meta-analysis. The results suggested that the DOR was not notably affected following sequential exclusion of each study in turn (Table IV). Secondly, the four studies that did not use ELISA as a test method were removed; a decreased pooled DOR (11.73; 95% CI, 6.58–20.92) was found, which suggested that the test assay may have had an effect on the results. No notable conclusion was drawn when the target population was limited to Chinese patients in a similar manner to that already described (pooled DOR, 16.29; 95% CI, 6.28–42.25). Statistical analysis was performed using Meta Disc (version 1.4) software.

## Discussion

A total of 11 studies were analyzed to evaluate the diagnostic accuracy of serum GP73 for HCC. The results demonstrated that GP73 is a useful marker as an independent diagnostic tool for HCC; however, multiple methodological limitations, a broad range of diagnostic accuracy values and heterogeneity were found in the included studies. Five of the studies reported that serum GP73 was superior to AFP as a serum marker (22–24,29,30), while the remaining six reported the opposite or had ambiguous results.

The potential biomarker for HCC investigated in the present study, serum GP73, is a 73-kDa transmembrane glycoprotein composed of 400 amino acids that normally resides in the epithelial cells of a range of human tissues (39). The presence of higher levels of serum GP73 in patients with hepatitis-B-virus-related HCC was first found by Block *et al* (13) in 2005. The detection of GP73 in the serum was based on its initial characterization as a resident Golgi membrane protein; however, it has been shown that GP73 cycles to the cell membrane for retrieval via a unique endosomal pathway (40). The results of such *in vitro* studies have demonstrated that GP73 can transiently be found at the plasma membrane, indicating a potential pathway for its release into the circulation. The mechanism underlying the upregulation of GP73 in HCC is yet to be elucidated, and further studies are required to investigate whether serum GP73 levels are also altered in patients with other types of solid tumor.

Western blotting, immunoblotting and ELISA are three of the main methods used to assay GP73, all of which exhibit certain disadvantages: The former two are semiquantitative and labor-heavy, while ELISA elicits disappointing results. In seven studies (25–30,32), the use of ELISA was unsuccessful at finding a significant elevation in serum GP73 levels in patients with HCC versus patients with liver cirrhosis. It has been suggested that GP73-specific serum autoantibodies may interfere with ELISA (10). Furthermore, several isoforms of GP73 corresponding with different patterns or levels of glycosylation have been found (41). Further investigation into whether the measurement of an HCC-specific GP73 isoform would improve the diagnostic accuracy is required.

Cancer comprises a diverse group of diseases that exhibit considerable differences in their etiology and biology; therefore, it is unlikely that a single biomarker would be able to detect all the types of cancer associated with a particular organ with sufficiently high specificity and sensitivity (11). The diagnostic value of GP73 in combination with AFP for HCC has been reported in seven studies (23,26–29,31,32), and the results were improved compared with those for a single marker.

Nine studies reported the diagnostic utility of serum GP73 by the stage of chronic liver disease and the conclusions were conflicting (22–24,26,28–32). In general, the GP73 level showed an increasing trend with the progression of liver disease. The results suggested that GP73 may be used as a serum marker for the diagnosis of liver diseases and for monitoring disease progression. It additionally appears that serum levels of GP73 in patients with HCC are not consistently affected by tumor size and differentiation, which may reflect the potential origin of HCC from cancer stem cells. If this finding is verified in further studies with large sample sizes, it may be beneficial for the early detection of HCC among the at-risk population.

The present study failed to find the reason for the existing heterogeneity within the studies. The most important factor contributing to this failure was that several of the studies investigating diagnostic accuracy lacked information on key elements of the study design and conduct. With incomplete and inaccurate reporting, it is not possible to correctly identify potential sources of bias and variability.

In conclusion, the present meta-analysis found that GP73 is a valuable marker as an independent diagnostic tool for HCC due to its high sensitivity and specificity. As such, GP73 may improve the detection and treatment of one of the most common global malignancies. Further studies are required to determine the effect of the etiology of the disease on the GP73 signal strength, the diagnostic accuracy of GP73 in detecting early HCC or cancer recurrence and the value of a combination of GP73 and AFP.

## Figures and Tables

**Table I:** Main characteristics of the studies included.

First author, year (ref.)	Country	TP/FP/FN/TN results (n/n/n/n)	Assay type	Cutoff value	HCC/cirrhosis/hepatitis/healthy/others (n/n/n/n/n)
Hu, 2010 (19)	China	24/15/7/78	Western blotting	7.4 RU	31/31/31/31/b
Mao, 2010 (20)	China/USA	589/89/200/3339	Immunoblotting	8.5 RU	789/512/337/1690/889
Marrero, 2005 (21)	China	99/21/45/131	Immunoblotting	10 RU	144/152/b/56/b
Morota, 2011 (22)	USA	62/61/8/98	ELISA	94.7 μg/l	70/35/52/72/b
Shi, 2011 (23)	China	50/5/23/102	ELISA	123.2 μg/l	73/13/32/62/b
Tian, 2011 (24)	China	115/46/38/49	ELISA	113.89 μg/l	153/95/115/109/b
Wang, 2009 (25)	USA	156/64/8/49	ELISA	NK	164/113/b/b/b
Xu, 2011 (26)	China	63/38/18/208	ELISA	NK	81/176^a^/40/b
Zhao, 2010 (27)	China	168/41/51/112	ELISA	100 ng/ml	219/110^a^/43/b
Wang, 2013 (28)	China	62/32/22/141	Immunoblotting	8.5 RU	84/80/32/61/b
Hou, 2013 (29)	China	58/16/21/58	ELISA	78.1 ng/l	84/80/32/61/b

a Data including both cirrhosis and chronic hepatitis.

b No data in this category.

HCC, hepatocellular carcinoma; RU, relative unit; TP, true-positive; FP, false-positive; FN, false-negative; TN, true-negative, NK, not known.

**Table II:** Summary judgments of the methodological quality of the included studies (QUADAS checklist).

QUADAS item	1	2	3	4	5	6	7	8	9	10	11
Representative patient spectrum?	Y	Y	Y	Y	Y	Y	Y	Y	Y	Y	Y
Selection criteria?	Y	Y	Y	Y	Y	Y	Y	Y	Y	Y	Y
Acceptable reference standard?	Y	Y	Y	Y	Y	Y	Y	Y	Y	Y	Y
Acceptable delay between tests?	UC	UC	UC	UC	UC	UC	UC	UC	UC	UC	UC
Partial verification avoided?	UC	Y	Y	UC	UC	Y	Y	UC	UC	Y	Y
Differential verification avoided?	UC	Y	Y	UC	UC	Y	Y	UC	UC	Y	Y
Incorporation avoided?	Y	Y	Y	Y	Y	Y	Y	Y	Y	Y	Y
Index test execution?	Y	Y	Y	Y	Y	Y	Y	N	Y	Y	Y
Reference standard execution?	N	Y	Y	N	N	Y	Y	N	N	Y	N
Reference standard results blinded?	N	N	N	N	N	N	N	N	N	Y	N
Index test results blinded?	Y	Y	Y	Y	Y	Y	Y	Y	Y	Y	Y
Relevant clinical information?	Y	Y	Y	Y	Y	Y	Y	Y	Y	Y	Y
Uninterpretable results reported?	Y	Y	Y	Y	Y	Y	Y	Y	Y	Y	Y
Withdrawals explained?	UC	UC	UC	UC	UC	UC	UC	UC	UC	UC	UC

UC, unclear; Y, yes; N, no; QUADAS, Quality Assessment of studies of Diagnostic Accuracy included in Systematic reviews.

**Table III:** Meta-regression of the effects of methodological characteristics on diagnostic accuracy.

Variable	Coefficient	Standard error	P-value	RDOR	95% CI
Assay	−0.117	0.4307	0.7930	0.89	0.33–2.40
Country	2.332	0.5633	0.0033	10.29	2.81–37.73
Year	−0.086	0.1850	0.6563	0.92	0.60–1.41

CI, confidence interval; RDOR, ratio of the diagnostic odds ratio.

**Table IV:** Changes in the DOR following sequential exclusion of each study in turn.

First author, year (ref.) of excluded study	Number of studies	DOR	95% CI
All studies included (19–29)	11	15.78	6.95–35.83
Hu, 2010 (19)	10	15.60	6.51–37.41
Mao, 2010 (20)	10	12.24	8.11–18.47
Marrero, 2005 (21)	10	16.01	6.49–39.44
Morota, 2011 (22)	10	16.15	6.70–38.92
Shi, 2011 (23)	10	14.34	5.99–34.33
Tian,2011 (24)	10	18.60	8.56–40.40
Wang, 2009 (25)	10	15.86	6.56–38.36
Xu, 2011 (26)	10	15.47	6.27–38.17
Zhao, 2010 (27)	10	16.73	6.87–40.72
Wang, 2013 (28)	10	16.17	6.61–39.53
Hou, 2013 (29)	10	16.51	6.86–39.71

DOR, diagnostic odds ratio; CI, confidence interval.
